# microRNA-155 inhibition restores Fibroblast Growth Factor 7 expression in diabetic skin and decreases wound inflammation

**DOI:** 10.1038/s41598-019-42309-4

**Published:** 2019-04-09

**Authors:** João Moura, Anja Sørensen, Ermelindo C. Leal, Rikke Svendsen, Lina Carvalho, Rie Juul Willemoes, Per Trolle Jørgensen, Håvard Jenssen, Jesper Wengel, Louise Torp Dalgaard, Eugénia Carvalho

**Affiliations:** 1 Center for Neuroscience and Cell Biology, University of Coimbra, Coimbra, Portugal; 2 Department of Science and Environment, Roskilde University, Roskilde, Denmark; 3 Faculty of Medicine, University of Coimbra, Coimbra, Portugal; 4 Nucleic Acid Center, Department of Physics, Chemistry and Pharmacy, University of Southern Denmark, Odense, Denmark; 5 Department of Geriatrics, University of Arkansas for Medical Sciences, Little Rock, Arkansas United States; 6 Arkansas Children’s Research Institute, Little Rock, Arkansas United States

## Abstract

Treatment for chronic diabetic foot ulcers is limited by the inability to simultaneously address the excessive inflammation and impaired re-epithelization and remodeling. Impaired re-epithelization leads to significantly delayed wound closure and excessive inflammation causes tissue destruction, both enhancing wound pathogen colonization. Among many differentially expressed microRNAs, miR-155 is significantly upregulated and fibroblast growth factor 7 (FGF7) mRNA (target of miR-155) and protein are suppressed in diabetic skin, when compared to controls, leading us to hypothesize that topical miR-155 inhibition would improve diabetic wound healing by restoring FGF7 expression. *In vitro* inhibition of miR-155 increased human keratinocyte scratch closure and topical inhibition of miR-155 *in vivo* in wounds increased murine FGF7 protein expression and significantly enhanced diabetic wound healing. Moreover, we show that miR-155 inhibition leads to a reduction in wound inflammation, in accordance with known pro-inflammatory actions of miR-155. Our results demonstrate, for the first time, that topical miR-155 inhibition increases diabetic wound fibroblast growth factor 7 expression in diabetic wounds, which, in turn, increases re-epithelization and, consequently, accelerates wound closure. Topical miR-155 inhibition targets both excessive inflammation and impaired re-epithelization and remodeling, being a potentially new and effective treatment for chronic diabetic foot ulcers.

## Introduction

Chronic diabetic foot ulceration (DFU) is one of the most debilitating complications of long-standing diabetes. DFU is, at least in part, a consequence of uncontrolled infection of foot wounds, due to the presence of neuropathy, peripheral vascular/arterial disease^1^, impaired angiogenesis and chronic low-grade inflammation^2^. Reduced blood flow restricts migration of leukocytes^3^, keratinocytes, fibroblasts and endothelial progenitor cells to the wounded site^4^. Long-term hyperglycemia promotes the activation of NFkB^5^, leading to chronic inflammation^2^ and impairing leukocyte activation and migration^3^. Despite the huge impact of long-term hyperglycemia on the progression of diabetes complications, the ACCORD and ADVANCE clinical trials^6^ showed that while glucose-lowering treatments reduce the risk of cardiovascular diseases, the risk of DFU and other complications still remains, thus, indicating that current therapies are not sufficient and there is an urgent need to identify better therapeutic interventions.

Several microRNAs (miRs) have been associated with DFU progression and severity^7^. While specific miRs, such as miR-31^8^, have been shown to improve wound healing, other miRs, such as miR-26a^9^, increase the severity of DFU. miR-155 fits in this second category^10–13^. It is a small (23 nucleotide), single-stranded, non-coding RNA originally identified as a gene on human chromosome 21, formerly called B-cell Integration Cluster^14^. The pre-miR-155 hairpin gives rise to two mature forms (miR-155-3p and miR-155-5p)^15^, where miR-155-5p is the major form (miRbase MI0000681, from now on referred as miR-155).

In murine models, whole-body over-expression of miR-155 leads to hypoglycemia, because miR-155 positively regulates insulin sensitivity and glucose uptake in insulin-sensitive cells, whereas complete deficiency of miR-155 results in hyperglycemia^11^. MiR-155 is expressed by immune cells^16^, including Th1 and Th17^17^, as well as other cells in inflammatory conditions^18^, and plays a pro-inflammatory role in cells, by targeting Cytotoxic T-Lymphocyte-associated protein (CTLA)- 4^19^, suppressor of cytokine signaling (SOCS)1, and SH2-Containing Inositol-5′-phosphatase (SHIP)1 from the Toll-Like Receptor (TLR)- 2 pathway^20^. Furthermore, inhibition of regulatory T cells by miR-155, both in control^17^ and diabetic subjects^13^, promotes the exacerbation of inflammation, which is involved in the pathology of psoriasis^17^. Moreover, anti-inflammatory drugs such as L-arginine and ibuprofen^12^, resveratrol^21^, vitamin D^22^ or M2000^23^ have been shown to downregulate miR-155.

MiR-155 is also important for the function of skin cells involved in wound healing, including keratinocytes^24^, dermal mesenchymal stem cells^25^, mast cells^26^, melanocytes^27^, adipocytes^28^ and fibroblasts^10^. Furthermore, miR-155 deficiency^29^ and miR-155 inhibition^30^ was shown to improve wound healing, in healthy and diabetic animal models, but the mechanism by which miR-155 impairs wound healing remains elusive.

In this work, we aimed to investigate the effect of specifically inhibiting miR-155 in diabetic skin on wound healing in a type 1 diabetic mouse model. Our diabetic mouse model confirmed a significant increase in miR-155 expression in the skin during wound healing and topical miR-155 inhibition improved wound closure via de-repression of FGF7 (fibroblast growth factor 7).

## Results

**Figure 1:**
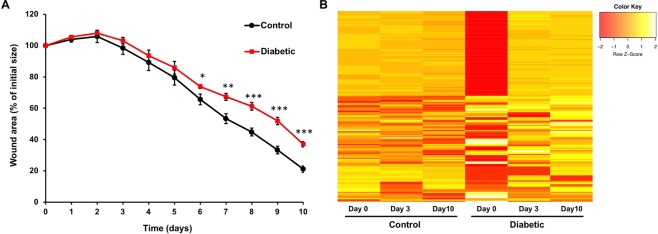
Diabetes significantly alters miR expression pattern and delays wound healing. (**A**) Wound size was assessed as percentage of initial size, along 10 days, in control and diabetic mice (n = 6 for each group). (**B**) Expression of 641 unique miRs, shown as heat map, in skin wound samples from the same control and diabetic mice, collected at baseline (day 0) and at days 3 and 10 post wounding. miR expression levels were normalized to the levels of U6 present on the arrays and control levels at day 0. MiRs changed by more than 2-fold (up or down) were included in the heat map. Data were analyzed using Student’s t-test; *p < 0.05; **p < 0.01; ***p < 0.001.

Wound healing is compromised under diabetic conditions, in humans^3^ and in rodents^31^. To confirm wound healing impairment, we used a well-established mouse model of diabetic wound healing^31^. Wound closure was monitored for 10 days post-wounding. Our results demonstrate wound healing impairment in diabetic mice (Fig. 1A). To evaluate the effect of diabetes on skin miR expression during wound healing, we used skin samples collected at baseline (Day 0) and Days 3 and 10 post-wounding and profiled miR expression. The array screening results indicate that in diabetic mice (n = 6 in each group – days 0, 3 and 10), a large fraction of the detected miRs show more than two-fold difference in expression at days 0, 3 or 10 following wounding (Fig. 1B): 36, 29 and 17 miRs were upregulated and 44, 35 and 37 miRs were downregulated in diabetic skin at these time points.

**Figure 2:**
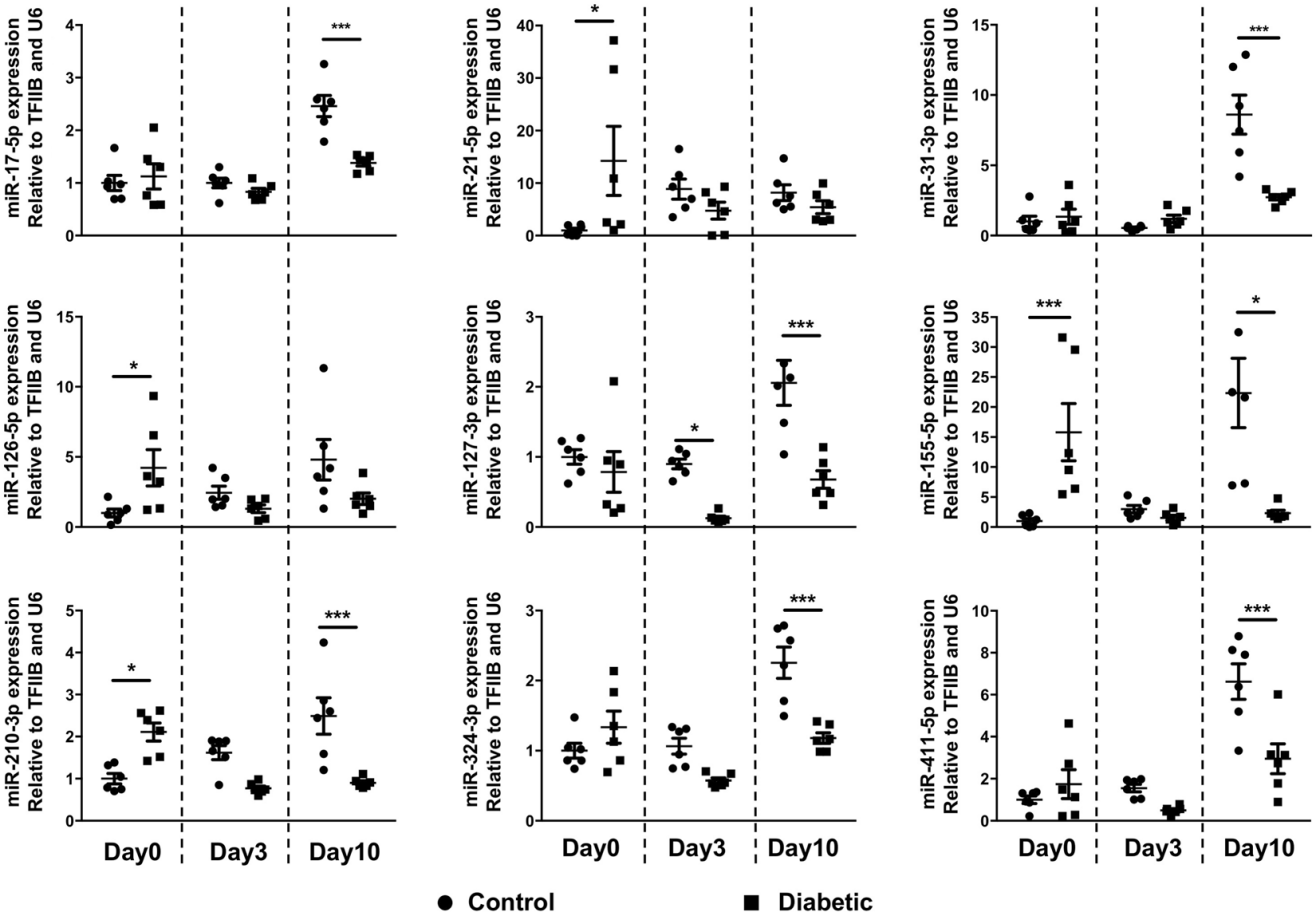
The expression of miR-155-5p and other miRs are significantly increase in diabetic mouse skin. The expression of selected miRs was confirmed by RT-qPCR and normalized to the mean of TFIIB and U6 levels and control baseline. miR-155-5p and miR-126-5p were significantly up-regulated in diabetic skin at baseline and miR-127-3p, miR-411-5p, miR-31-3p and miR-31-5p are significantly down-regulated in diabetic wound skin at day 10 post-wounding. Data were analyzed by two-way ANOVA with Dunnets’ post hoc correction; *p < 0.05; ***p < 0.001.

Array results were further validated by RT-qPCR (reverse transcription quantitative polymerase chain reaction) for individual targets (Fig. 2). Interestingly, while miR-155 was significantly overexpressed in diabetic mouse skin, compared to non-diabetic mice (15.8 fold ± 4.8 vs 1.0 ± 0.4, p < 0.001) at baseline, it was markedly decreased in diabetic skin (2.3 ± 0.5 vs 22.3 ± 5.8, p < 0.05) in the later phases of wound healing (Day 10). Similar to miR-155, miR-126-5p was increased in diabetic skin at Day 0 and suppressed at Day 10 compared with non-diabetic skin. A number of other miRs were significantly decreased in diabetic skin wounds at Day 10: miR-17-5p, miR-31-3p, miR-31-5p, miR-324-3p and miR-411-5p, while miR-127-3p was downregulated at Day 3 and 10 in diabetic wounded skin. miR-21-5p was increased at Day 0 in diabetic skin, but not at other time points.

**Figure 3:**
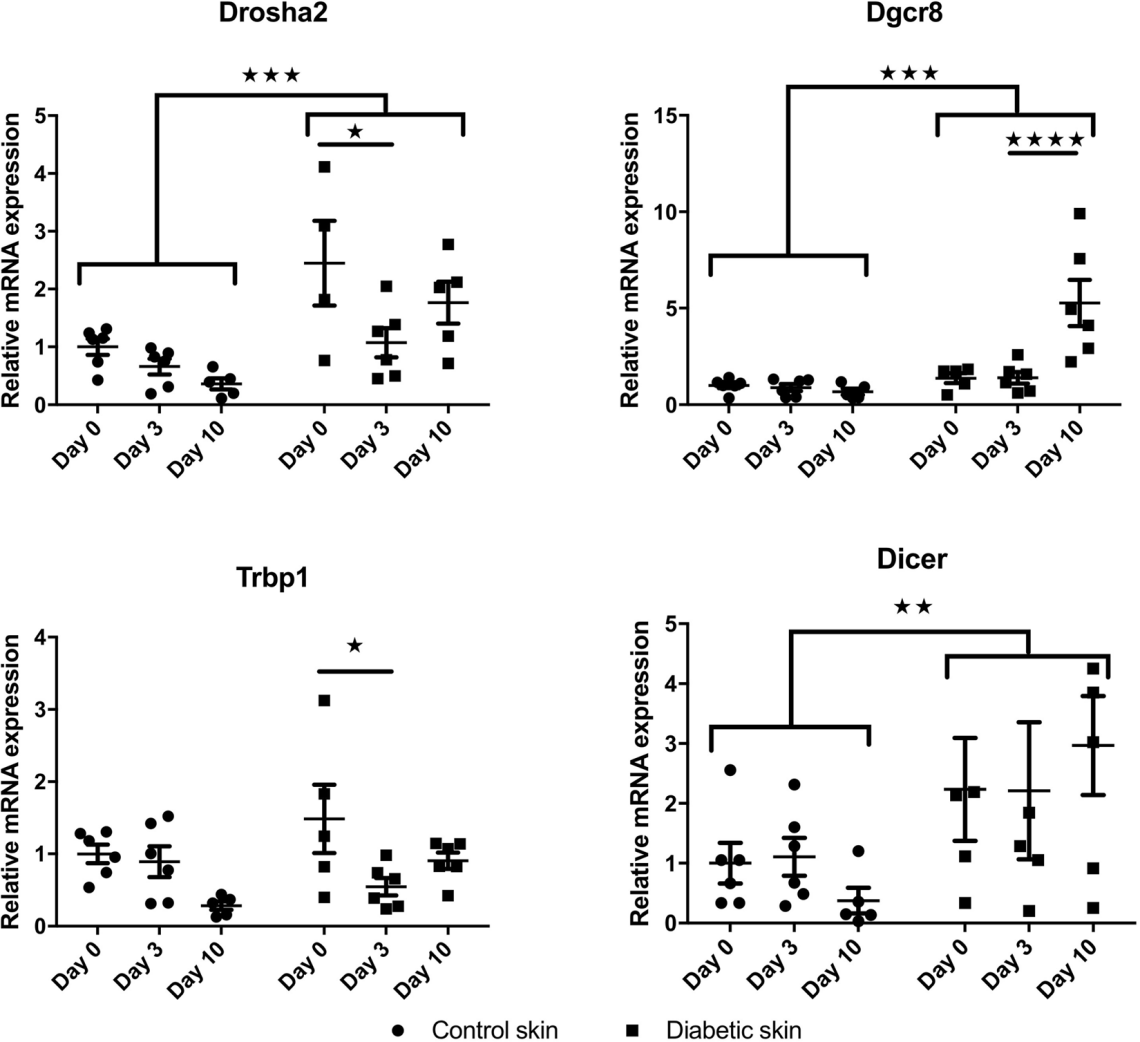
Diabetes significantly increases the expression of proteins involved in miR processing. Expression of Drosha2 (**A**), DiGeorge Syndrome Chromosomal Region 8 (DGCR8) (**B**), TAR RNA Binding Protein 1 (TRBP-1) (**C**) and Dicer (**D**) in skin wound samples from control and diabetic mice (n = 6 for each group), collected at baseline (day 0) and at days 3 and 10 post wounding. mRNA levels were quantified by q-RT-PCR and normalized to the mean of TFIIB and U6 levels and control baseline. Data were analyzed by two-way ANOVA with Dunnet’s post hoc correction; *p < 0.05; **p < 0.01; ***p < 0.001; ****p < 0.0001.

Since a large fraction of the analyzed miRs were decreased in diabetic mouse skin, we further evaluated the mRNA expression levels of four proteins involved in miR processing, TAR RNA Binding Protein 1 (Trbp-1), DiGeorge Syndrome Chromosomal Region 8 (Dgcr8), Dicer and Drosha-2 (Fig. 3). Interestingly, mRNA levels of *Drosha-2*, *Dgcr8* and *Dicer* were significantly increased (p < 0.001 for *Drosha-2* and *Dgcr8* and p < 0.01 for Dicer) in diabetic mouse skin, before and after wound induction, when compared to non-diabetic mice. While the overall miR expression was decreased at Day 0 in diabetic mice, the increased RNA transcript level of these factors involved in miR processing (*Drosha-2*, *Dgcr8, Dicer, Trbp1*) may suggest an unaccommodated feedback from decreased miR action in diabetic mouse skin at Day 0.

**Figure 4:**
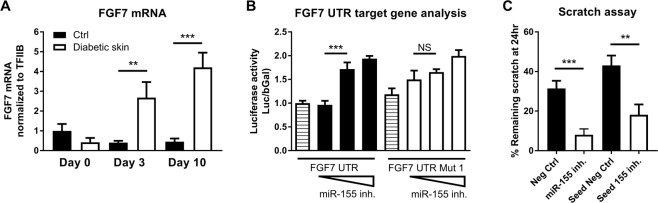
miR-155 inhibitor significantly increases FGF7 expression and improves keratinocyte migration *in vitro* and *in vivo*. (**A**) FGF7 mRNA levels in skin wound samples from control and diabetic mice (n = 6/group), collected at baseline (Day 0) and at days 3 and 10 post wounding, quantified by RT-qPCR and normalized to the mean of TFIIB and U6 levels and control baseline. (**B**) Luciferase activity of HaCAT cells transfected with a luc-reporter vector carrying the FGF7 3′ untranslated region (UTR) or with a mutated vector where one (of two) miR-155 binding sites is mutated and 12, 25 or 35 pmol/48 well miR-155 inhibitor oligo. (**C**) HaCAT cells were transfected with miR-155 inhibitor or scrambled miR (neg control). Scratches were made 48 h post-transfection and measured again 24 h later. Seed 155i and Seed Neg: LNA-oligonucleotides binding to seed region of miR-155 or a negative control. Data were analyzed using two-way ANOVA with Dunnet’s post hoc correction (**A**) and Student’s t-test (**B**,**C**); **p < 0.01; ***p < 0.001.

Since miR-155 was one of the most differentially expressed miRs in diabetic and control skin and it has previously been shown to be important for wound healing^29^, we chose to investigate miR-155 in depth. Importantly, we also evaluated the expression of one *in silico* identified target of miR-155’s, FGF7 (also known as keratinocyte growth factor) (Fig. 4A). As opposed to control mice, where FGF7 mRNA levels decrease during wound healing, in diabetic mouse skin, the levels of FGF7 mRNA inversely correlate with the expression of miR-155, increasing significantly during wound healing. To test miR-155 efficacy in downregulating FGF7 expression, we constructed an FGF7-luciferase-reporter vector containing the 3′ UTR (untranslated region) of the FGF7 gene ((FGF7 UTR) (Fig. 4B) and analyzed luciferase activity in response to different concentrations of miR-155 inhibitor in human HaCaT keratinocytes. FGF7 UTR mediated reporter-gene activity increased (FGF7 UTR: 1.65-fold ± 0.06 at 25 pmol inhibitor/well and 1.99-fold ± 0.12 at 35 pmol inhibitor/well compared with control, both p < 0.001) with miR-155 inhibition in a dose-dependent manner. Importantly, removing just one of the predicted two miR-155 target sites of the FGF7 UTR significantly decreases the response to miR-155 inhibition (Fig. 4B).

Furthermore, we performed an *in vitro* scratch-assay to test the effect of miR-155 on cell migration. Transfection of HaCaT keratinocyte cells with full-length miR-155 inhibitor significantly increased *in vitro* scratch closure, in hyperglycemic conditions (Fig. 4C): While negative control transfected cells had 31.4% ± 3.9% remaining scratch after 24 hrs, miR-155 inhibitor transfected cells had only 8.1% ± 2.9% remaining (p < 0.0001). Moreover, scratch closure was also significantly enhanced when using a shorter inhibitor only binding to the seed site of miR-155 (Seed 155 inhibitor) (Seed Neg. Ctrl.: 43.1% ± 5.0% vs Seed 155 inhibitor:18.2% ± 5.2%) (Fig. 4C and Suppl. Fig. 1).

**Figure 5:**
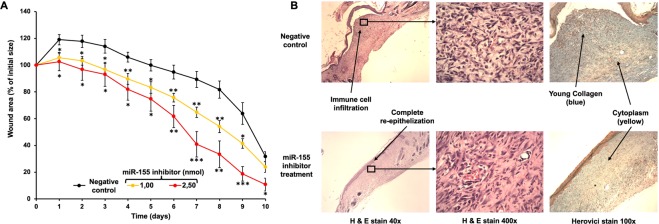
Topical administration of miR-155 inhibitor reduces inflammation and significantly improves wound healing kinetics in diabetic mice. (**A**) Wound size was assessed as percentage of initial size, along 10 days, in diabetic mice treated topically with 2.5 nmol of Neg. Ctrl. oligo and 1.00 and 2.50 nmol of miR-155 inhibitor (n = 6 for each group). (**B**) Representative images of Hematoxylin-Eosin staining and Herovici staining of mouse wound skin tissue of diabetic mice treated with Neg. Ctrl. Oligo (control) and 2.50 nmol of miR-155 inhibitor, collected at Day 10 post-wounding (n = 6 for each group with one representative example shown). Data were analyzed using Student’s t-test; *p < 0.05; **p < 0.01; ***p < 0.001 vs Neg. Ctrl. oligo.

While the scratch wound *in vitro* assay is a simple wound healing model and does not reflect the multiple cellular interactions taking place in *in vivo*, we performed wound healing experiments in diabetic mice. To test the *in vivo* effect of miR-155 inhibition we measured wound closure kinetics over a period of 10 days (Fig. 5A). Dorsal wounds were induced on the back of diabetic mice and were subsequently topically treated with different concentrations of the miR-155 inhibitor, twice a day, until Day 3 (n = 6 in each group). Topical administration of the miR-155 inhibitor after wound induction significantly improved wound closure, especially with 2.5 nmol dose applied. Improvement in wound closure was visible as early as the first day of treatment and was persistent even after the 3 days of treatment until the end of the experiment, 10 days post-wounding. The 0.25 nmol dose had no effect on wound healing and 2.5 nmol and 10 nmol doses had a similar effect (data not shown). Moreover, the negative control inhibitor did not display altered wound healing kinetics compared with the saline control (data not shown), showing that the action of the miR-155 inhibitor is very likely to be sequence specific.

**Figure 6:**
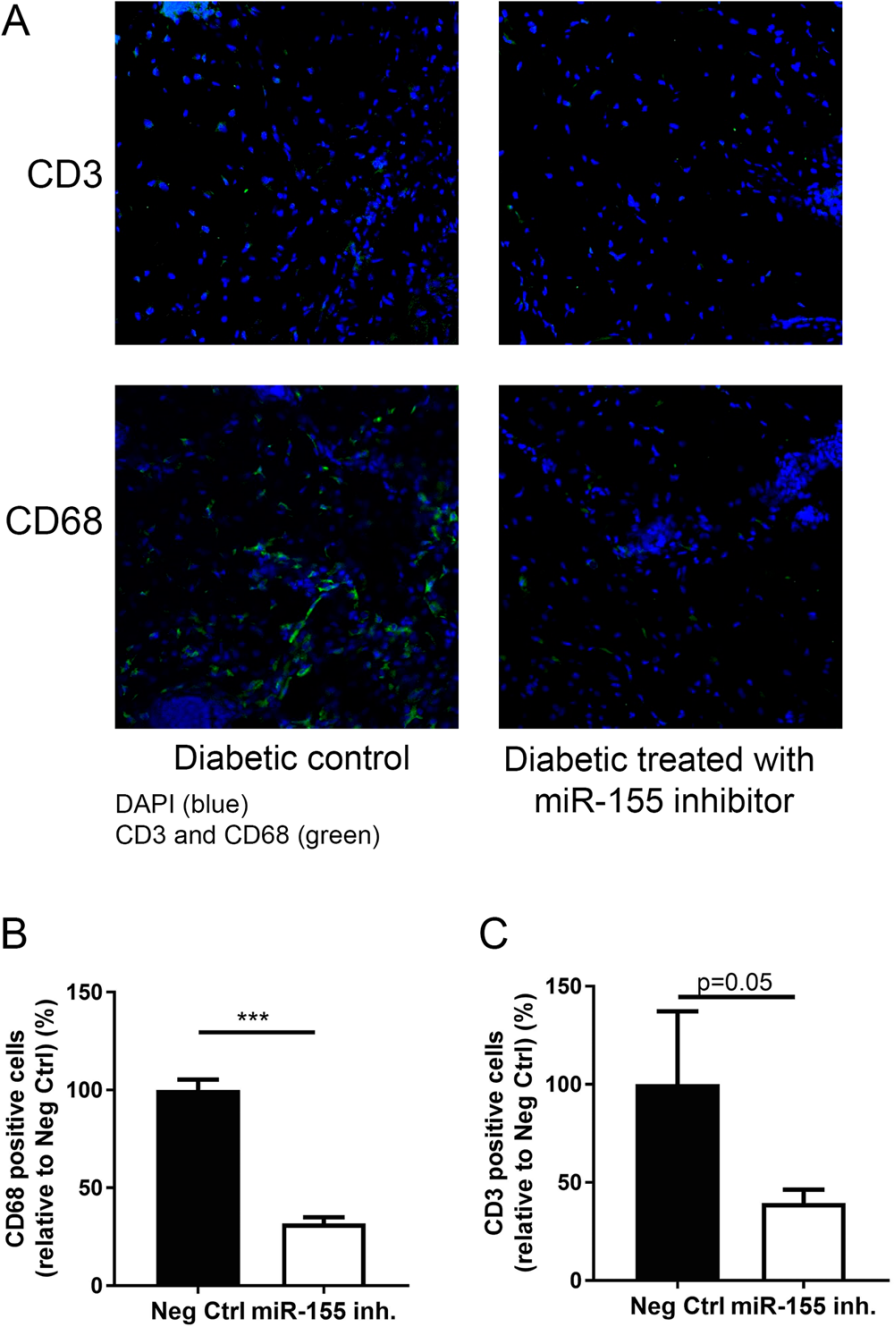
Topical administration of miR-155 inhibitor reduces T-cell and macrophage wound infiltration in diabetic mice. (**A**) Representative fluorescence microscopy images of T-cells (CD3^+^) and macrophages (CD68^+^) in wound skin tissue of diabetic mice treated with 2.5 nmol Neg. Ctrl. oligo (control) or 2.5 nmol of miR-155 inhibitor, collected at day 10 post-wounding (n = 3 for each group with one representative example per group shown). (**B**) Quantifications of the number of CD68^+^ macrophages and (**C**) Quantifications of the number of CD3^+^ T-cells. Blue – DAPI, Green – CD3 or CD68. The results were analyzed using student’s t-test. ***p < 0.001 vs Neg. Ctrl. oligo.

HE and Herovici’s staining at Day 10 post-wounding indicated anti-inflammatory effects of *in vivo* miR-155 inhibition (Fig. 5B). As opposed to diabetic mouse wounds treated with the negative inhibitor, where a clear immune cell infiltration was observed, diabetic mouse wounds treated with 2.5 nmol of miR-155 inhibitor presented a complete re-epithelization, with no traces of inflammation. We also observed a prevalence of young, unorganized collagen in control wounds (Fig. 5B), in clear contrast with miR-155 inhibitor treated wounds, where young collagen expression was residual, indicating that miR-155 inhibition accelerates wound maturation. To further evaluate immune cell infiltration at Day 10 post-wounding, we performed fluorescent immunohistochemical staining for CD3, to identify T-cells and CD68 to identify macrophages (Fig. 6) on control and miR-155 inhibitor treated wounds (n = 3 in each group). Macrophage wound infiltration was significantly decreased (32% of Ctrl.) in diabetic wounds treated with 2.5 nmol of miR-155 inhibitor in control diabetic wounds than (Ctrl.: 77.7 ± 3.1 vs MiR-155 inhibitor: 24.7 ± 1.8 cells/field; p < 0.0001) (Fig. 6B), while T-cell infiltration was 39% of Ctrl. following inhibition of miR-155 (Ctrl.: 22.0 ± 6.0 vs MiR-155 inhibitor: 8.7 ± 1.1 cells/field, p = 0.05) (Fig. 6C).

**Figure 7:**
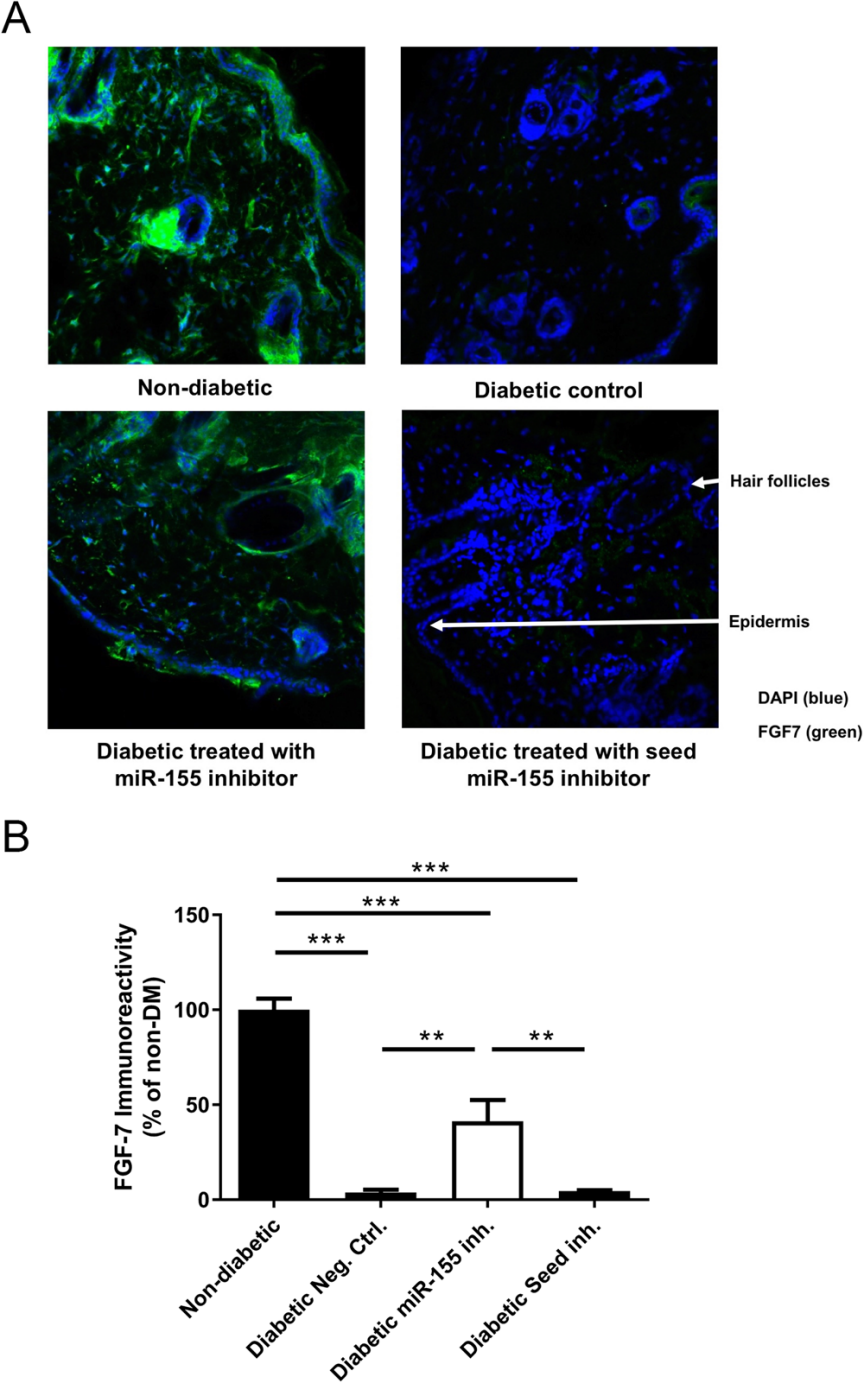
Topical administration of miR-155 inhibitor restores FGF7 expression. (**A**) Representative images of FGF7 protein expression assessed by fluorescence microscopy on mouse wound skin tissue of non-diabetic (n = 4) and diabetic mice treated with Neg. Ctrl. oligo (control, n = 3), 2.5 nmol of miR-155 inhibitor (n = 3) or 2.5 nmol of Seed miR-155 inhibitor (n = 3), collected at day 10 post-wounding. Seed miR-155 inhibitor: LNA-oligonucleotides binding to seed region of miR-155. Blue – DAPI, Green – FGF7. (**B**) Quantification of FGF7 expression. Data are presented as mean ± SD of the percentage of non-diabetic condition. Data were analyzed using one-way ANOVA with Dunnet’s post hoc correction; **p < 0.01, ***p < 0.001 vs non-diabetic.

To elucidate the action of the miR-155 inhibitor on FGF7 expression, we performed fluorescent immunohistochemical staining for FGF7 (Fig. 7A), on samples collected at Day 10 post-wounding. We stained non-diabetic wounded skin and observed high levels of FGF7 associated with hair shafts, as well as staining throughout sub cutis and the epidermis. In diabetic skin, treated with the negative control inhibitor oligo, FGF7 levels were clearly suppressed (3.8% ± 2.5%, p < 0.001 compared to non-diabetic control), both in the hair shaft follicles and in the epidermis. Treatment with miR-155 inhibitor markedly increased FGF7 levels in hair shafts, in the subcutis and in the epidermis of diabetic mice (41.3% ± 19.6%, p < 0.01 compared to diabetic mice)(Fig. 7B). Thus, FGF7 is clearly de-repressed at the protein level by miR-155 inhibition, while the shorter Seed miR-155 inhibitor had no effect *in vivo*, under the tested conditions.

We tested the action of the miR-155 inhibitor on FGF7 mRNA levels in skin from diabetic mice wounded and subsequently topically treated with inhibitor. Despite clear functional actions of the miR-155 inhibitor at Day 3, there was no effect of the miR-155 inhibitor on steady state FGF7 mRNA level or on other predicted mRNA targets (*Ctla4*, *Hbp1*, *Stat3*, *Nfia*) (data not shown). Similarly, despite clear effects on scratch-assay migration (Suppl. Fig. 1), the miR-155 inhibitor did not alter measured miR-155 levels or FGF7 mRNA levels (data not shown). We also evaluated the effect of miR-155 inhibitor on angiogenesis, by staining the vessels for CD31, and we did not observe changes between the negative control oligo and the miR-155 inhibitor (Suppl. Fig. 2).

## Discussion

Skin injury triggers acute inflammatory responses, beginning with recruitment of neutrophils and monocytes to the site of injury that, in turn, secrete various inflammatory cytokines, chemokines and growth factors coordinating wound repair^32^. Excessive inflammation observed in DFU causes tissue damage through the release of increasing levels of various proteins involved in bacterial control, such as granzymes and perforins, that degrade the extracellular matrix^33^ and inhibit re-epithelization^34^ impairing wound healing. This excessive inflammation is partially controlled by miRs, including miR-155^35^, and its suppression, either directly using a miR-155 inhibitor^36^ or indirectly using other substances^21,37^, has an anti-inflammatory effect in different conditions.

Our results show that miR-155 is over-expressed in diabetic mouse skin, when compared to non-diabetic control mice. Most importantly, we show, for the first time, that FGF7 mRNA expression is significantly decreased during wound healing under diabetes conditions, contrary to what is observed in wound healing in healthy mice. Moreover, we demonstrate that topical application and inhibition of miR-155 for only 3 days has long-lasting and positive effects on diabetic wound healing.

Diabetic individuals show decreased levels of miR-155 expression in peripheral blood plasma^11,38^ and mononuclear cells^39^, as well as in the kidney, heart, aorta and sciatic nerve^40^, when compared to age and sex-matched controls. However, miR-155 was shown to be increased in peripheral blood of diabetic patients with retinopathy^13^ and in retinal cells of diabetic rats^41^. Moreover, circulating miR-155 levels are down-regulated in pre-diabetic individuals^38^ and this decrease is not reversed by insulin treatment and glycemic control^42^. Variations between tissues or related to concomitant diabetes-associated pathologies may explain the differences in miR-155 expression observed in these studies, reinforcing the need to characterize miR-155 expression in the various tissues and conditions.

It has been reported that miR-155 has pro-inflammatory effects and that miR-155 inhibition leads to a reduction in inflammation, observed by decreased IL-1β and TNF-α levels^43^, and more regular collagen fiber arrangement^43^ and faster diabetic wound healing in a rat model^30^. Non-diabetic mice lacking miR-155 show improved wound repair^29^ through increase in M2 macrophage polarization and increase in type I collagen deposition. Accordingly, increased miR-155 expression is associated with M1 polarization which, in turn, has a negative effect on diabetic wound healing^44^. Our results also demonstrate that topical miR-155 inhibition reduces T-cell and macrophage wound infiltration, consequently reducing tissue inflammation and promotes collagen fiber rearrangement, accelerating wound maturation in diabetic wounds. It will be relevant to further explore the role of miR-155 on inflammatory responses and the extracellular matrix composition during wound healing.

Moreover, our results show, for the first time, that miR-155 impairs diabetic wound re-epithelization, by targeting FGF7, which is essential for keratinocyte migration, proliferation and consequently wound closure^45^. Early studies have shown that FGF7 over-expression favors wound healing in various ways that are not restricted to keratinocyte or fibroblast proliferation and migration, but also involve increased revascularization and antimicrobial effects^46^. Here, we demonstrate that topical inhibition of miR-155 leads to increased keratinocyte migration and faster wound closure, in a dose-dependent manner. We also show *in vitro* that miR-155 regulates keratinocyte migration through inhibition of the FGF7 3′ untranslated region (UTR).

However, when evaluating the temporal modulation of miR-155 and FGF7 it is apparent that other factors than miR-155 are likely to contribute to the regulation of FGF7 mRNA and protein levels. For example, in diabetic animals miR-155 peaks at day 0 and is significantly less expressed at day 10, while no difference in FGF7 mRNA abundance is observed at day 0 between diabetics and controls, and FGF7 transcript is significantly more abundant at day 10 in diabetics. When considering other miRNAs as regulators then miR-21 also has a target site in the FGF7 3′UTR, which may contribute to suppression of FGF7 protein at day 0 in diabetic skin (where miR-21 is upregulated). Moreover, other factors than microRNAs (such as inflammatory cytokines^47^) control FGF7 mRNA and protein amounts. In addition, inhibition by microRNAs on their mRNA or protein targets is not instantaneous but rather slow, and the temporal effect of a microRNA also depends on the turnover of the protein, providing a possible explanation for discrepant observations between the miR-155 and FGF7 levels.

The FGF7 mRNA levels and FGF7 immunostainings in diabetic skin at day 10 following wounding did not correlate, but the mRNA of FGF7 in diabetic skin could be increased in a compensatory response. Not all microRNAs act to cause complete degradation of their cognate mRNA targets, but instead primarily halts the translation of the mRNA into protein. In fact, we could not detect significant increase in the FGF7 mRNA by treatment with the miR-155 inhibitor (data not shown), despite clear increases at the FGF7 protein levels and responses of the FGF7 3′UTR to miR-155 inhibitor in reporter assays.

Our results suggest that topical administration of miR-155 inhibitors, at the wound site, may have significant therapeutic value in DFU treatment, especially if applied during the first days after wounding, where miR-155 inhibition will have an immunosuppressive effect and decrease tissue damage.

## Methods

### Multiple low-dose streptozotocin diabetic mouse model

C57BL/6 male mice (25–30 g) obtained from Charles River Corporation Inc. (Barcelona, Spain) were housed at 22–24 °C with a 12 hrs light/dark cycle with *ad libitum* access to water and food. Experimental protocols were in accordance with the European Community law for Experimental Animal studies (86/609/CEE; 2007/526/CE) and were approved by the Institutional (Organ Responsible for Animal Welfare of the Center for Neurosciences and Cell Biology and Faculty of Medicine of the University of Coimbra) and Governmental (Directorate-General for Food and Veterinary of the Portuguese Ministry of Agriculture) Research Ethical Boards.

Diabetes was induced as previously described^31^. Briefly, streptozotocin (STZ) (50 mg/kg) in saline solution was injected *i.p*., for 5 consecutive days. Seven days post STZ injection, blood glucose was measured to confirm the diabetic phenotype. Mice with blood glucose levels above 250 mg/dL (Accu-Chek glucometer, Roche, Basel/Switzerland), were considered diabetic. Animals were treated with isophane (NPH) insulin (0.1–0.2 units), subcutaneously, as needed, to avoid weight loss. Animals were kept diabetic for 6 weeks prior to the wounding experiments. Like diabetic patients, STZ-induced diabetic mice also develop chronic low-grade inflammation leading to wound healing impairment^31^.

### Wound healing model and treatments

Wound induction was performed as previously described^31^. Briefly, control or diabetic mice were anesthetized with Ketamine/Xylazine (100/10 mg/kg, *i.p.)*. After removing the dorsal hair, two 6 mm excisional wounds 2 cm apart were created using a punch biopsy tool (Miltex, Rietheim-Weilheim, Germany). The wound area was traced daily onto acetate paper to follow rates of wound closure for 10 days post-wounding. Wound size was determined with ImageJ version 1.46 (NIH Image, USA).

Diabetic mice were used for miR-inhibitor treatments. Both wounds were treated topically, twice daily up to day 3 post wounding, with a miR-155 inhibitor (5′-TcaCaaTuaGcaTuaA-3′) (0.25, 1, 2.5 or 10 nmol) or with a negative control oligo (5′-CaaTagGguCaaGauT-3′); locked nucleic acid (LNA) bases are written in capital letters while 2′-*O*-methyl RNA bases in small letters. Oligos were designed using LNA bases for every third nucleotide and the backbone was phosphorothioate substituted for enhanced binding strength and stability. A seed miR-155 inhibitor (5′-AGCAuTaA-3′) was synthesized to only target the seed sequence of miR-155, and a seed control oligo (5′-TCAAgAuT-3′) was also synthesized^48^. Animals were sacrificed at days 3 or 10 post-wounding and the wounded skin was harvested for analysis.

### Skin wound homogenization and RNA extraction

Skin tissue (50–100 mg) was homogenized with 1 mL TRI Reagent (Sigma Aldrich, St. Louis, Missouri, USA) with a polytron homogenizer followed by purification as per manufacturers’ instructions. The RNA pellet was dissolved in DEPC water (50 µL). RNA concentration and purity were assessed using the NanoDrop ND-1000 spectrophotometer (ThermoFisher Scientific, Waltham, Massachusetts, USA). Samples were stored at −80 °C until further analysis.

### Profiling miRs in skin wounds

MiR expression was measured on pools of skin samples using Rodent TaqMan Low Density Array cards, v.2.0 for RT-primer pool A and v3.0 for RT-primer pool B, containing 641 unique murine miRs (ThermoFisher Scientific, Waltham, Massachusetts, USA) according to manufacturer’s instructions. Each pool contained six samples from the same experimental group, with total RNA input of 600 ng per array card. Briefly, total RNA was reverse-transcribed using multiplex RT primer pool sets followed by quantitative PCR (qPCR) step with sequence-specific primers and probes on the TaqMan® MicroRNA Arrays. Expression data were obtained using the Viia 7 qPCR system (ThermoFisher Scientific, Waltham, Massachusetts, USA). Data was normalized against the stably expressed U6 snRNA available on the array and relative miR expression was calculated using the comparative ∆∆Ct method (2^−∆∆Ct^), against the expression of the same miR in control mice at day 0.

### Measurement of miR and mRNA levels by RT-qPCR

miRs were detected using reverse-transcription quantitative PCR (RT-qPCR) as previously described^49^. Oligonucleotides used for reverse transcription and qPCR are shown in Supplementary Table 1. Fibroblast growth factor (FGF) 7 mRNA levels were quantified using qPCR primers (QT00172004, Qiagen, Hilden/Germany), with Quantitect Sybr 2x Master Mix (Qiagen, Hilden, Germany) in 10 µL reactions using the MX3005 qPCR system (Agilent, Santa Clara, California, USA). Transcripts were quantified using standard curve quantification, diluted skin cDNA served as input to generate the standard curve. The geometric mean of transcription factor (TF)IIB and U6 levels were used to normalize for variation in input template. The geometric mean of these two transcripts was unaltered.

### Cell culture and transfections

Human clonal keratinocyte (HaCaT) cells were cultured in Dulbeccos Modified Eagle Medium (DMEM, 20 mM glucose) (ThermoFisher Scientific, Waltham, Massachusetts, USA) supplemented with FBS (10%) and Penicillin/Streptomycin (1%). MiR-155 levels were measured by RT-qPCR and this microRNA is expressed in HaCaT cells although at moderate levels, with Ct levels from 1 ng cDNA starting at cycle 25–27. For scratch migration assays, HaCaT cells were seeded 30.000 cells per well in 48 well plates and allowed to adhere for 24 hrs, before transfection with miR inhibitors. Transfections consisted of inhibitor (25 pmol) and Lipofectamine 2000 (0.5 µL) (ThermoFisher Scientific, Waltham, Massachusetts, USA) per well and were prepared according to manufacturer instructions in triplicate. After 24 hrs the medium was changed to DMEM containing glucose (20 mM) supplemented with FBS (10%) and Penicillin/Streptomycin (1%) and scratches were performed. Following washes in medium to remove non-adherent cells, microscope images were acquired at time zero and 24 hrs later. Distance between scratch edges was calculated using ImageJ and are presented as percentage of remaining scratch after 24 hrs.

Wild type and mutant FGF7 3′ untranslated region (UTR) constructs for miR-155-5p site 1 were cloned by PCR amplification of mouse genomic DNA (oligonucleotides listed in Tab. 1) and inserted into the XbaI and FseI sites in the 3′ UTR of the luc2 gene of pGL4.13 (Promega, Madison, Wisconsin, USA).

For reporter-gene analysis of 3′ UTR reporter constructs, HaCaT cells were seeded in 48 well plates (20.000 cells per well) and allowed to adhere for 24 hrs. Following media change, transfections consisting of 35 ng beta-gal expression plasmid and 315 ng luciferase UTR reporter-gene plasmid with or without miR inhibitor (amounts indicated in figures) were made using linear polyethylineimine (PEI25) (Sigma Aldrich, St. Louis, Missouri, USA)^50^. After 24 hrs cells were lysed and luciferase measured using the DualLight Assay (Perkin Elmer, Waltham, Massachusetts, USA) on a GloMax96 instrument with a dual-injector system (Promega, Madison, Wisconsin, USA). Transfections were made in triplicate and luciferase activity was normalized to beta galactosidase activity to control for differences in cell transfection rate.

### Immunofluorescence staining and histological analysis

Samples from frozen optimal cutting temperature compound (OCT) blocks were sectioned with a 10 µm thickness, put on glass slides and kept at −20 °C for later use. Thawed skin sections were incubated with anti-FGF7 (1:100) (PA5-49715, Invitrogen, Carlsbad, California, USA), anti-CD3 (1:100) (PC630, Merck Millipore, Darmstadt, Germany), anti-CD68 (1:100) (ab955, Abcam, Cambridge, UK) or anti-CD31 (1:200)(Merck Millipore, Darmstadt, Germany) and the nuclei were stained with DAPI (Sigma Aldrich, St. Louis, Missouri, USA). After washing with PBS, sections were incubated with Alexa fluor 488 conjugated goat antiserum against rabbit (1:250) (Invitrogen, Carlsbad, California, USA) or Alexa fluor 594 conjugated goat antiserum against rat (1:500). The sections were then imaged using a confocal (FGF7, CD3 and CD68) or fluorescence (CD31) microscope. FGF7 was quantified by measuring the fluorescence intensity of two independent microscopy fields with 200x magnification and T-cells (CD3^+^), macrophages (CD68^+^). The CD31 was measured by counting the number of vessels from ten images of three different sections (200x magnification). Hematoxylin and Eosin (HE) (Merck Millipore, Darmstadt, Germany) and Herovici’s (American Mastertech Scientific, Lodi, California, USA) stainings were performed in 3 µm thickness paraffin sections, according to the manufacturer’s instructions. The sections were imaged using a transmission microscope.

### Statistics

GraphPad Prism and Excel were used for statistical analysis. Significance was tested by Students t-test (for 2 groups), repetitive, paired t-test (*in vivo* wound healing analysis) or one or two-way ANOVA with Dunnets’ post hoc correction (multiple groups) with a significance level of p < 0.05. Data shown are averages from replica experiments.

## Supplementary information

Supplementary data1
