# Supplementary material for: microRNA-155 inhibition restores Fibroblast Growth Factor 7 expression in diabetic skin and decreases wound inflammation
Source: Sci Rep. 2019 Apr 9;9:5836. doi: 10.1038/s41598-019-42309-4 (PMC6456606; doi:10.1038/s41598-019-42309-4)
Supplement: Supplementary file 1 — Supplementary data1 [file 41598_2019_42309_MOESM1_ESM.pdf]

Title

microRNA-155 inhibition restores Fibroblast Growth Factor 7 expression in diabetic skin and decreases wound inflammation

Author list

João Moura, Anja Sørensen, Ermelindo C. Leal, Rikke Svendsen, Lina Carvalho, Rie Juul Willemoes, Per Trolle Jørgensen, Håvard Jenssen, Jesper Wengel, Louise Torp Dalgaard, Eugénia Carvalho

Supplementary Figure 1

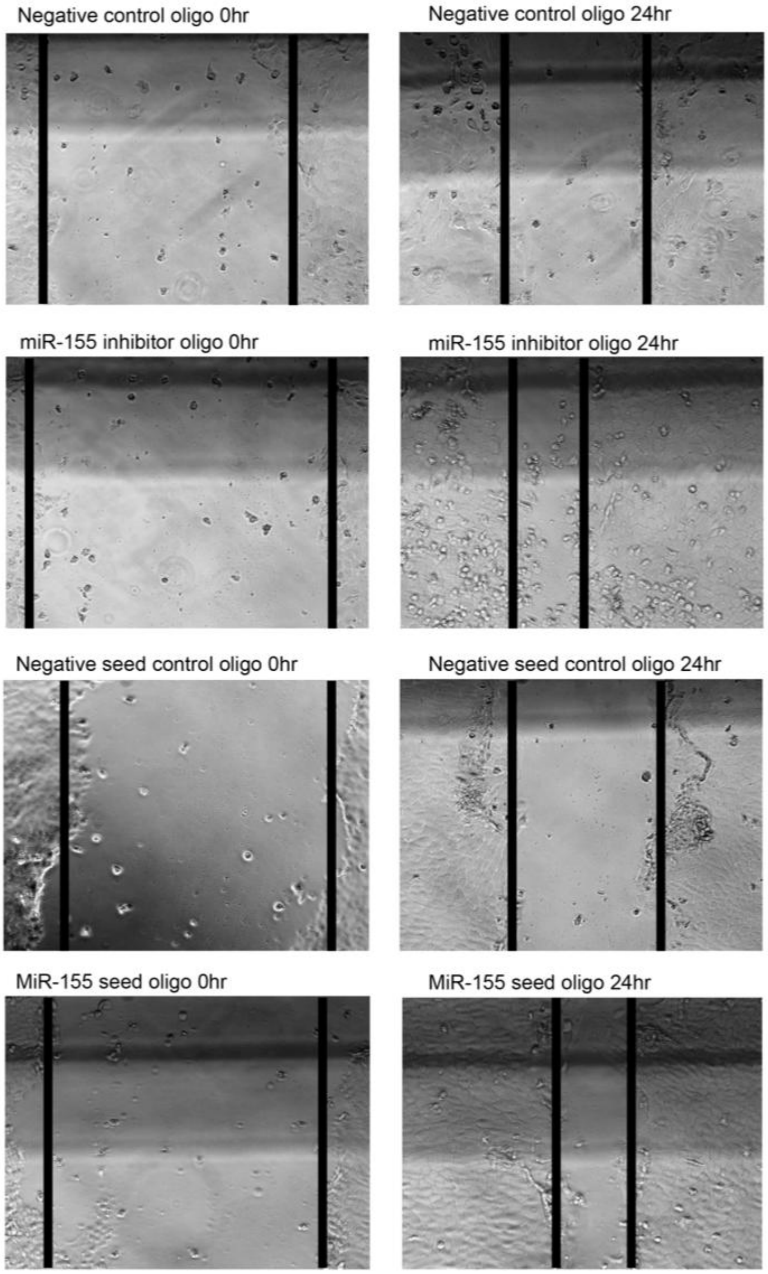

Suppl. Fig. 1. Representative phase contrast microscope images from HaCaT scratch migration assays. For scratch migration assays, HaCaT cells were seeded in plates and allowed to adhere for 24hrs, before transfection with miR inhibitors. After 24hrs the medium was changed and scratches were performed. Following washes in medium to remove non-adherent cells, microscope images were acquired at time zero and 24hrs later. Distance between scratch edges was calculated using ImageJ and are presented as percentage of remaining scratch after 24hrs and averages are presented in Figure 4C.

Supplementary Figure 2

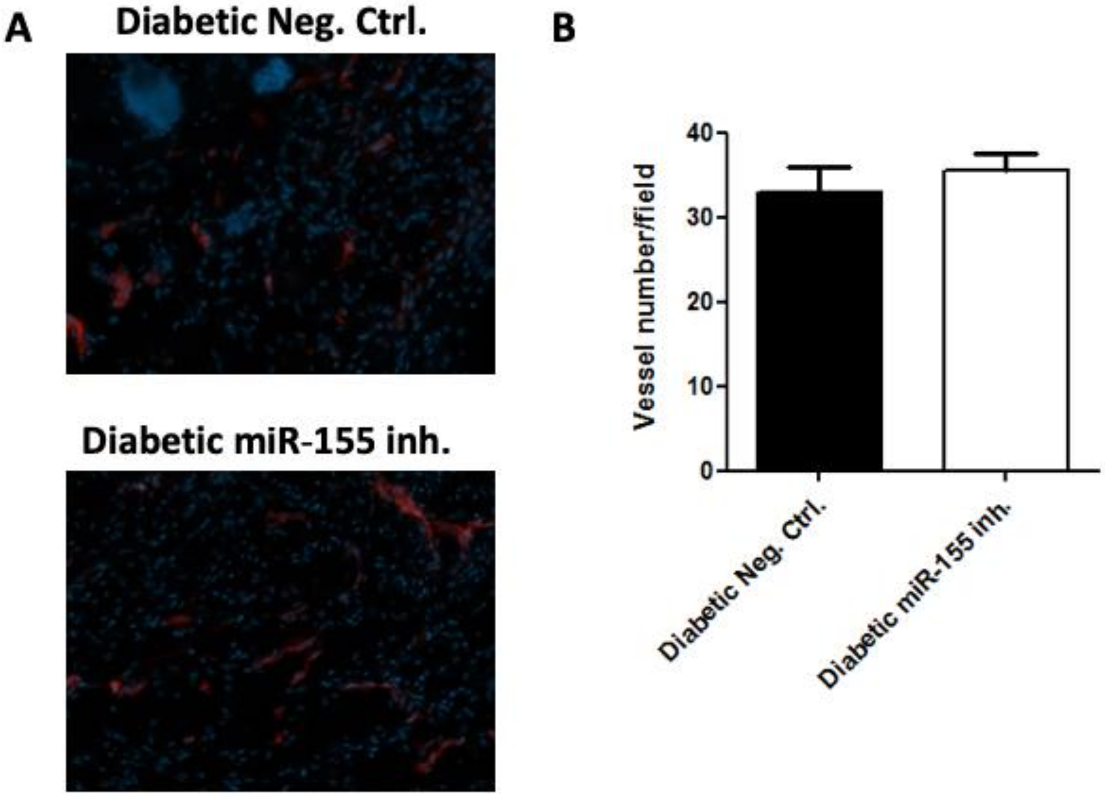

Suppl. Fig. 2. Effects of topical inhibition of miR-155 on CD31+ positive endothelial cells. (A) Representative fluorescence microscopy images of CD31+ endothelial cells in wound skin tissue of diabetic mice treated with 2.5 nmol Neg. Ctrl. oligo (control) or 2.5 nmol miR-155 inhibitor, collected at day 10 post-wounding (n=3 for each group with one representative example per group shown). (B) Quantifications of the number of CD31+ endothelial cells. Blue – DAPI, Red – CD31. The results were analyzed using student's t-test.

Supplementary table 1 - Oligonucleotide sequences used for RT-qPCR and cloning

microRNA	Type	Oligo sequence
miR-31-5p	Reverse	5’CTCAACTGGTGTCGTGGAGTCGGCAATTCAGTTGAGCAGCTATG
	Forward	5’ACACTCCAGCTGGGAGGCAAGATGCTGGCA
miR-210-3p	Reverse	5’CTCAACTGGTGTCGTGGAGTCGGCAATTCAGTTGAGTCAGCCGC
	Forward	5’ACACTCCAGCTGGGCTGTGCGTGTGACAGC
miR-409-3p	Reverse	5’CTCAACTGGTGTCGTGGAGTCGGCAATTCAGTTGAGAGGGGTTC
	Forward	5’ACACTCCAGCTGGGGAATGTTGCTCGGTGA
miR-324-3p	Reverse	5’CTCAACTGGTGTCGTGGAGTCGGCAATTCAGTTGAGCCAGCAGC
	Forward	5’ACACTCCAGCTGGGACTGCCCCAGGTGC
miR-411-5p	Reverse	5’CTCAACTGGTGTCGTGGAGTCGGCAATTCAGTTGAGCGTACGCT
	Forward	5’ACACTCCAGCTGGGTAGTAGACCGTATAG
miR-31-3p	Reverse	5’CTCAACTGGTGTCGTGGAGTCGGCAATTCAGTTGAGGATGGCAA
	Forward	5’ACACTCCAGCTGGGTGCTATGCCAACATATT
miR-93-5p,<br>miR-106-5p,<br>miR-17-5p	Reverse	5’CTCAACTGGTGTCGTGGAGTCGGCAATTCAGTTGAGCTACCTGC
miR-93-3p	Forward	5’ACACTCCAGCTGGGCAAAGTGCTGTTCGTGC
miR-106-5p	Forward	5’ACACTCCAGCTGGGCAAAGTGCTAACAGTGC
miR-17-5p	Forward	5’ACACTCCAGCTGGGCAAAGTGCTTACAGTGC
miR-127-3p	Reverse	5’CTCAACTGGTGTCGTGGAGTCGGCAATTCAGTTGAGAGCCAAGC
	Forward	5’ACACTCCAGCTGGGTCGGATCCGTCTGAGC
miR-503-5p	Reverse	5’CTCAACTGGTGTCGTGGAGTCGGCAATTCAGTTGAGCTGCAGTA
	Forward	5’ACACTCCAGCTGGGTAGCAGCGGGAACAGTA
miR-188-5p	Reverse	5’CTCAACTGGTGTCGTGGAGTCGGCAATTCAGTTGAGCCCTCCAC
	Forward	5’ACACTCCAGCTGGGCATCCCTTGCATGGT
miR-146a-5p	Reverse	5’CTCAACTGGTGTCGTGGAGTCGGCAATTCAGTTGAGAACCCATG
	Forward	5’ACACTCCAGCTGGGTGAGAACTGAATTCCA
MiR-126-5p	Reverse	5’CTCAACTGGTGTCGTGGAGTCGGCAATTCAGTTGAGCGCGTACC
	Forward	5’ACACTCCAGCTGGGCATTATTACTTTTGG
miR-155-5p	Reverse	5’CTCAACTGGTGTCGTGGAGTCGGCAATTCAGTTGAGACCCCTAT
	Forward	5’ACACTCCAGCTGGGTTAATGCTAATTGTGAT
miR-29a	Reverse	5’ACACTCCAGCTGGGTAGCACCATCTGAAAT
	Forward	5’CTCAACTGGTGTCGTGGAGTCGGCAATTCAGTTGAGTAACCGAT
miR-21-5p	Reverse	5’CTCAACTGGTGTCGTGGAGTCGGCAATTCAGTTGAGTCAACATC
	Forward	5’ACACTCCAGCTGGGTAGCTTATCAGACTGA
URP universal	Reverse	5’GTTCTGCTCCAACCTTTGCCT
TFIIB	Forward	5’GTTCTGCTCCAACCTTTGCCT
	Reverse	5’TGTGTAGCTGCCATCTGCACTT
U6	Reverse	5’AACGCTTCACGAATTTGCGT
	Forward	5’CTCGCTTCGGCAGCACA
Dicer	Forward	5’TGCTGCAGTAAGCTGTGCTA
	Reverse	5’CATTGGTGAGGAAGCAGGGG
Dgcr8	Forward	5’CGAGCCACCCTGGAAATTCT
	Reverse	5’GATGTGGTTAAAATACTCCAGTTCT

Exp5	Forward	5’TGTCACATACAAGCCTGCGT
	Reverse	5’AGTCCTGGGGGCCTTACTTT
Drosha 1	Forward	5’TTTAATTCCCGGGGCTTCCT
	Reverse	5’CCTGCAGGATTCACAGTCTCTAC
Drosha 2	Forward	5’CGGGACTCTCTAGACTGTGA
	Reverse	5’GCTACATCTTCCGCTCACGA
Tarbp1	Forward	5’ACGGAGGAGGGAATGAGTGA
	Reverse	5’CTGAAGAAGGCTGATCGGGG
Tarbp2	Forward	5’GAGGCTGTAGTCACGGTGG
	Reverse	5’TCTAGGAGAGAAAAAGAACTGGG
Ago4	Forward	5’CGCTTCAGCGCCAATATTCC
	Reverse	5’CGAATTGGTTTCCCAACGGT
FGF7 UTR	Forward	5’ACGT TCTAGA AAACCAGTTTCATTCAGCAGGGA
FGF7 UTR WT	Reverse	5’ACGTGGCCGGCCAGTTTAATGCTGTAATCACAGAGGG
FGF7 UTR mut 1	Reverse	5’ACGTGGCCGGCCAGTTTTTAGCtGtAATCACAGAGGG
FGF7 UTR mut 2	Forward	5’CTTCAAATCTTTCTAGCTAAAAGTCTTTTAAAAT
	Reverse	5’ATTTTAAAAGACTTTTAGCTAGAAAGATTTGAAG
